# Extracorporeal Membrane Oxygenation (ECMO) for Refractory Cardiac Arrest

**DOI:** 10.21980/J88W69

**Published:** 2020-10-15

**Authors:** Kevin Hanneken, David Gaieski, Amrita Vempati, Ronald Hall

**Affiliations:** * Thomas Jefferson University Hospital, Department of Emergency Medicine, Philadelphia, PA

## Abstract

**Audience:**

Our target audience includes emergency medicine residents/physicians.

**Introduction:**

Treating cardiac arrest is a common theme during simulated emergency medicine training; however, less time is focused on treating *refractory* cases of cardiac arrest. There are varying definitions of refractory cardiac arrest, but it is most commonly defined as the inability to obtain return of spontaneous circulation (ROSC) after 10–30 minutes of appropriate cardiopulmonary resuscitation (CPR).^1^,^2^ More specifically, refractory ventricular fibrillation (VF) is defined as VF persisting despite 3 shocks, or the combination of 3 unsuccessful shocks plus amiodarone.^1^,^3^ Extracorporeal Membrane Oxygenation (ECMO) is becoming an increasingly utilized tool in the emergency department for severe cases of both pulmonary and cardiovascular pathology, and has been shown to be successful in cases of refractory cardiac arrest. Using ECMO in this scenario is known as Extracorporeal Cardiopulmonary Resuscitation (ECPR), referring to the emergent implementation of veno-arterial (VA) ECMO, and data have shown significantly improved neurologically-intact survival compared to routine CPR.^3^–^7^

**Educational Objectives:**

Our objectives go beyond the basics of advanced cardiac life support (ACLS), forcing the learner to think about alternative treatments for refractory cardiac arrest. By the end of this session, the learner should be able to:

1. Recognize refractory cardiac arrest and realize when advanced management is required beyond the basics of ACLS
2. Recite the indications/contraindications to ECMO
3. Differentiate the physiology and clinical requirements between using venous-venous (VV) ECMO for respiratory failure, and using VA ECMO for cardiovascular failure
4. Identify the anatomical cannulation sites for VV vs VA ECMO
5. Perform the procedural skills to cannulate for both VV and VA ECMO

**Educational Methods:**

This simulation is flexible. We used a high-fidelity mannequin with the “Endo-Circuit” to practice cannulating for ECMO, but the learning objectives can still be achieved with a lower-fidelity mannequin and cannulation device. The “Endo-Circuit” is a novel, low-cost vascular model developed by Dr Tomoyuki Endo from Sendai, Japan to practice ECMO cannulation.^8^,^9^

**Figure:**
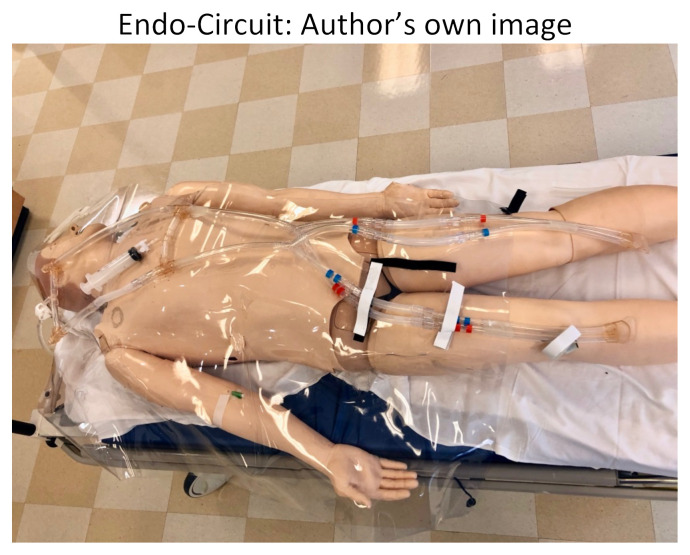
Endo-Circuit: Author’s own image

Alternatively, a lower-fidelity model can be utilized if the Endo-Circuit is not available. We recommend using clear silicone tubing, which can be found at your local hardware store. This tubing should be at least 12mm in internal-diameter to accommodate the large ECMO catheters. We cut the tubing into 6-inch pieces so they could easily be swapped out for multiple participants to practice cannulating, all in a cost-effective manner. Red and blue tape was applied to differentiate the artery from the vein.

**Figure:**
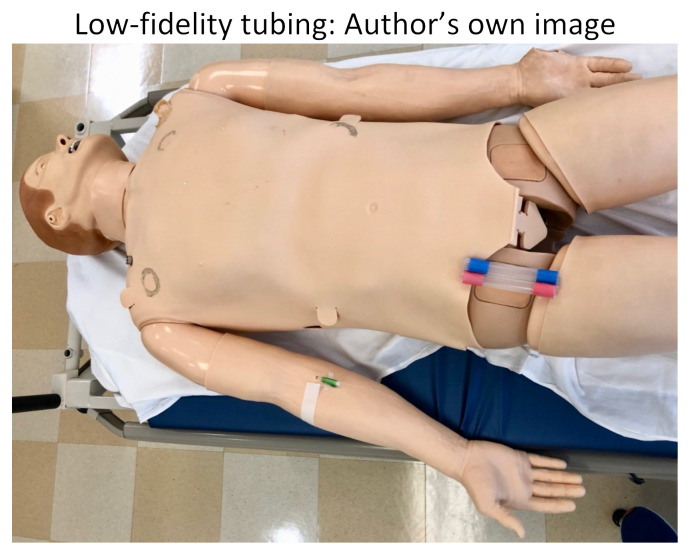
Low-fidelity tubing: Author’s own image

We split our educational session into different stages. The first stage included the high-fidelity mannequin without the Endo-Circuit because we did not want to reveal our ultimate goal of starting the patient on ECMO by having the tubing overlying the mannequin. Neither standard ACLS methods nor advanced medications for refractory cardiac arrest lead to achieving ROSC in this scenario. Stage 1 ends when the learners suggest starting the patient on ECMO and call the appropriate consultants. After a short debrief on stage 1, we then transition to a 2^nd^ mannequin that we had in the back of the room. This mannequin had the Endo-Circuit overlying, and everything was covered with a sheet, again so as not to reveal the goal of the simulation from the beginning. On this 2^nd^ mannequin, we practiced cannulating for VA ECMO in the setting of cardiac arrest. Below are photos of the ECMO cannulation kit, the cannulated Endo-Circuit, as well as the cannulated lower-fidelity silicone tubing.

**Figure:**
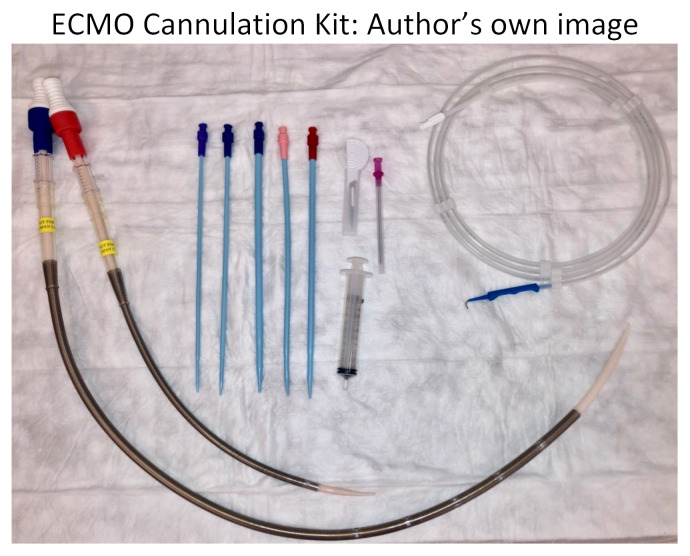
ECMO Cannulation Kit: Author’s own image

**Figure:**
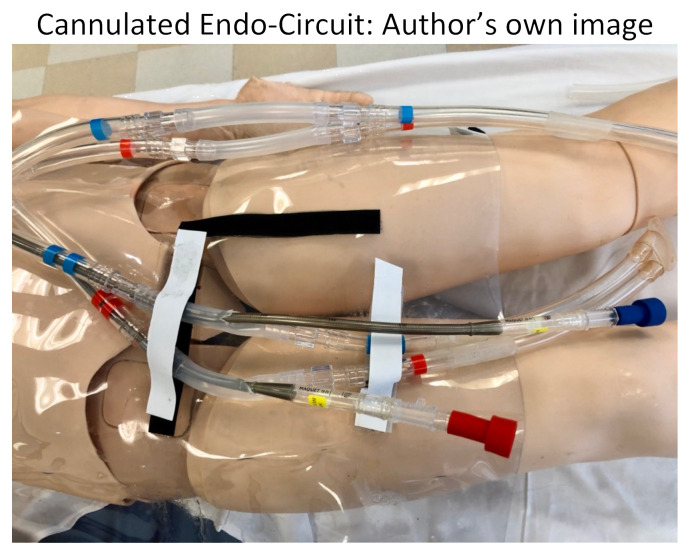
Cannulated Endo-Circuit: Author’s own image

**Figure:**
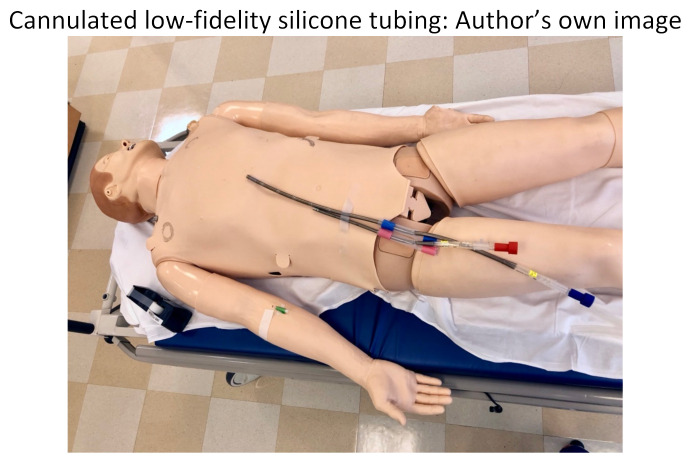
Cannulated low-fidelity silicone tubing: Author’s own image

**Research Methods:**

The learners filled out a post-simulation survey, which included questions specifically focused on the educational objectives (as mentioned above). We used a 1–5 Likert scale ranging from strongly disagree (1) to strongly agree (5) to quantify how the residents’ understanding of the learning objectives improved after the simulation. This survey also included questions taken directly from the Debriefing Assessment for Simulation in Healthcare (DASH), which is a validated evaluation tool developed by the Center for Medical Simulation (CMS) for evaluating the efficacy of the educational content.^10^ The DASH scoring system involves a 7-point scale ranging from extremely ineffective/detrimental (1) to extremely effective/outstanding (7).

**Results:**

Thirty-one resident-learners participated in the simulation, and we received 22 survey responses. All of the learning objectives obtained a mean score >4 out of 5, with the exception of improving the learners’ differential diagnosis for refractory cardiac arrest, which received a mean score of 3.86. The most successful of the learning objectives was improving the learners’ procedural skills for ECMO cannulation, which received a mean score of 4.68. The DASH questions also reflected the success of the simulation, with 3 of the 6 questions receiving a mean score >6 out of 7, and the other 3 questions receiving a score >5.

**Discussion:**

According to this data, the learners found the simulation to be effective in expanding their knowledge base and improving procedural skills for starting critically-ill patients in refractory cardiac arrest on ECMO. Practicing the cannulation procedure on the Endo-Circuit was shown to be the most useful aspect of this simulation. The DASH survey questions further demonstrate that our methods created an engaging, structured environment to identify knowledge gaps and simultaneously fill them using hands-on, active learning.

**Topics:**

Extracorporeal membrane oxygenation, ECMO, cardiac arrest, refractory cardiac arrest, V fib, ventricular fibrillation, CPR, cardiopulmonary resuscitation, ECPR, extracorporeal cardiopulmonary resuscitation, ACLS, advanced cardiac life support, HOCM, hypertrophic obstructive cardiomyopathy, critical care, emergency medicine.

## USER GUIDE

**Table:** 

List of Resources:	
Abstract	29
User Guide	34
Instructor Materials	38
Operator Materials	47
Debriefing and Evaluation Pearls	51
Simulation Assessment	54

**Learner Audience:**

Senior residents and attendings

**Time Required for Implementation:**

Preparation: 1 hour to review the case/debriefing topics; 1 hour to set up the mannequin and other equipment (possibly longer if you are going to use the Endo-Circuit). Time for case: *10–15 minutes for stage 1 of running the code; 20–30 minutes for demonstrating/practicing ECMO cannulation*.

Time for debriefing: *10–20 minutes*

**Recommended Number of Learners per Instructor:**

3–5 participants per case

**Topics:**

ECMO, extracorporeal membrane oxygenation, cardiac arrest, refractory cardiac arrest, V fib, ventricular fibrillation, CPR, cardiopulmonary resuscitation, ECPR, extracorporeal cardiopulmonary resuscitation, ACLS, advanced cardiac life support, HOCM, hypertrophic obstructive cardiomyopathy, critical care, emergency medicine.

**Objectives:**

By the end of this simulation session, the learner will be able to:

1. Recognize refractory cardiac arrest and realize when advanced management is required beyond the basics of ACLS
2. Recite the indications/contraindications to ECMO
3. Differentiate the physiology and clinical requirements between using VV ECMO for respiratory failure and using VA ECMO for cardiovascular failure
4. Identify the anatomical cannulation sites for VV vs VA ECMO
5. Perform the procedural skills to cannulate for both VV and VA ECMO

### Linked objectives and methods

This simulation is split into 4 stages. Stage 1 involved the learners running the code using the ACLS algorithm, identifying this to be a case of refractory ventricular fibrillation, and thus forcing them to think beyond the ACLS algorithm, eventually reaching for other options in their clinical tool-belt such as ECMO. Stage 1 was aimed at fulfilling Objective 1 above. Once the learners suggest starting ECMO and call the appropriate consultants, the simulation is paused, allowing time to debrief the code (this is Stage 2). This leads into Stage 3, which involves transitioning to the 2^nd^ mannequin with the Endo-Circuit and/or other cannulation device. During Stage 3, the instructor introduces Objectives 2–5, briefly going through the indications/contraindications to starting ECMO, explaining the physiology behind VV vs VA ECMO, and then demonstrating, step by step on the mannequin, how to perform cannulation for the different ECMO scenarios. Stage 4 involves active, hands-on learning by having the trainees perform the cannulation procedure, while thinking about the anatomy of the cannulation sites (venous vs arterial), to help reinforce the physiology behind ECMO, and gain a better understanding of the indications to utilize VV vs VA strategies in differing clinical scenarios.

Starting a patient on ECMO is clearly a high-stakes scenario, and it is inappropriate for a learner to practice initiation for the first time during a real patient-encounter. This simulation helps to build the foundational knowledge of when and how to initiate a patient on ECMO and allows the learners to apply this knowledge using hands-on procedural learning by practicing the appropriate cannulation techniques. All of this is accomplished in a controlled, low-risk, engaging learning environment. The goal is to allow the learners to make mistakes and improve upon their clinical skills during the simulation, so that they are better prepared to take care of critically-ill patients while on shift in the real world.

### Recommended pre-reading for instructor

The following resources were useful to prepare for the simulation:

- Sweeney B. Extracorporeal Membrane Oxygenation (ECMO) in the ED. EM Docs. Published Jan. 2015 Available at: www.emdocs.net/extracorporeal-membraneoxygenation-ecmo-in-the-ed/.
- Ghobrial M, Nugent K. A Review of ECMO in the ED: History, Mechanics, Common Indications, and Future Implications. EMRA. Aug. 2019. Available at: www.emra.org/emresident/article/ecmo-in-the-ed/. Lu CK, Holtz M, Donaldson R, O’Brien M. Extracorporeal Membrane Oxygenation. WikEM, Oct. 2019. Available at: wikem.org/wiki/Extracorporeal_membrane_oxygenation.
- Weingart, S. Podcast 057 – Resuscitative Extra-Corporeal Life Support (ECMO). EMCrit Blog. Published September 26, 2011. Available at: https://emcrit.org/emcrit/ecmo/.
- Weingart S. EMCrit 265 – ECPR 2.0 (ECMO CPR). EMCrit Blog. Published on February 5, 2020. Available at: https://emcrit.org/emcrit/ecpr-2-0/.

### Learner responsible content

- The learners would benefit from the same resources provided for the instructors, noted above.

### Results and tips for successful implementation

31 resident-learners participated in the simulation, and we received 22 post-simulation survey responses (71% response rate). The first 5 questions aimed to evaluate *the educational content* of the simulation. We used a 1–5 Likert scale ranging from “strongly disagree (1)” to “strongly agree (5)” to measure how well the simulation improved the following clinical and procedural skills:

Question 1: The simulation session helped improve my **differential diagnosis** for refractory cardiac arrest.

5) Strongly Agree: 1
4) Agree: 18
3) Neutral: 2
2) Disagree: 1
1) Strongly Disagree: 0
Mean Score: 3.86

Question 2: The simulation session helped improve my **medical management** for refractory cardiac arrest.

5) Strongly Agree: 7
4) Agree: 13
3) Neutral: 2
2) Disagree: 0
1) Strongly Disagree: 0
Mean Score: 4.22

Question 3: The simulation session helped improve my understanding of the **indications/contraindications for ECMO**.

5) Strongly Agree: 10
4) Agree: 12
3) Neutral: 0
2) Disagree: 0
1) Strongly Disagree: 0
Mean Score: 4.45

Question 4: The simulation session helped clarify when to use **veno-venous (VV) vs veno-arterial (VA)** ECMO.

5) Strongly Agree: 9
4) Agree: 10
3) Neutral: 3
2) Disagree: 0
1) Strongly Disagree: 0
Mean Score: 4.27

Question 5: The simulation session helped improve my **procedural skills** regarding how to **cannulate** for ECMO.

5) Strongly Agree: 15
4) Agree: 7
3) Neutral: 0
2) Disagree: 0
1) Strongly Disagree: 0
Mean Score: 4.68

The next 6 questions were taken directly from the DASH questionnaire (Debriefing Assessment for Simulation in Healthcare), and were focused on assessing *the methodology* of the simulation in achieving the learning objectives. The DASH scoring system involves a 7-point scale ranging from “extremely ineffective/detrimental (1)” to “extremely effective/outstanding (7).”

Question 1: The instructor set the stage for an engaging learning experience:

7) Extremely Effective/Outstanding: 9
6) Consistently Effective/Very Good: 10
5) Mostly Effective/Good: 3
4) Somewhat Effective/Average: 0
3) Mostly Ineffective/Poor: 0
2) Consistently Ineffective/Very Poor: 0
1) Extremely Ineffective/Detrimental
Mean Score: 6.27

Question 2: The instructor maintained an engaging context for learning

7) Extremely Effective/Outstanding: 12
6) Consistently Effective/Very Good: 7
5) Mostly Effective/Good: 3
4) Somewhat Effective/Average: 0
3) Mostly Ineffective/Poor: 0
2) Consistently Ineffective/Very Poor: 0
1) Extremely Ineffective/Detrimental: 0
Mean Score: 6.41

Question 3: The instructor structured the debriefing in an organized way

7) Extremely Effective/Outstanding: 7
6) Consistently Effective/Very Good: 10
5) Mostly Effective/Good: 4
4) Somewhat Effective/Average: 1
3) Mostly Ineffective/Poor: 0
2) Consistently Ineffective/Very Poor: 0
1) Extremely Ineffective/Detrimental: 0
Mean Score: 6.05

Question 4: The instructor provoked in-depth discussions that led me to reflect on my performance

7) Extremely Effective/Outstanding: 5
6) Consistently Effective/Very Good: 11
5) Mostly Effective/Good: 5
4) Somewhat Effective/Average: 1
3) Mostly Ineffective/Poor: 0
2) Consistently Ineffective/Very Poor: 0
1) Extremely Ineffective/Detrimental: 0
Mean Score: 5.91

Question 5: The instructor identified what I did well or poorly, and why

7) Extremely Effective/Outstanding: 5
6) Consistently Effective/Very Good: 7
5) Mostly Effective/Good: 7
4) Somewhat Effective/Average: 3
3) Mostly Ineffective/Poor: 0
2) Consistently Ineffective/Very Poor: 0
1) Extremely Ineffective/Detrimental: 0
Mean Score: 5.64

Question 6: The instructor helped me see how to improve or how to sustain good performance

7) Extremely Effective/Outstanding: 4
6) Consistently Effective/Very Good: 10
5) Mostly Effective/Good: 7
4) Somewhat Effective/Average: 1
3) Mostly Ineffective/Poor: 0
2) Consistently Ineffective/Very Poor: 0
1) Extremely Ineffective/Detrimental: 0
Mean Score: 5.77

Overall, these results indicate a high level of success in achieving our learning objectives through the simulation. The learners indicated that the most useful part of the simulation session was the hands-on aspect of practicing the cannulation techniques for ECMO. The lowest score among the first 5 questions related to the differential diagnosis of refractory cardiac arrest. Therefore, more time could have been spent on this topic during the debrief session during Stage 2 of the stimulation. The DASH questions further validate our educational methods for the simulation, achieving greater than 6/7 for the first 3 questions, and greater than 5/7 for the next 3 questions.

If we were to do this simulation again, we would recommend having the learners perform both a pre-survey, as well as a post-survey, and to have the learners complete these at the time of the training, which will help increase the number of participants who filled out the survey.

## Supplementary Information



## Figures and Tables

**Table:** 

#1	Cardiac monitor showing Vfib throughout the entire case^11^
#2	Echo: fibrillations, thick septum consistent with HOCM, no pericardial effusion, left ventricle larger than right ventricle^12^
#3	Lab work will not significantly change management in this simulation, but the following lab values are available if the learners ask for them: complete blood count, electrolytes, extended electrolytes, venous blood gas, cardiac/coags, biliary, toxicology

**Table:** 

**Vitals:**	Heart Rate (HR) 0 Blood Pressure (BP) N/A	
Respiratory Rate (RR) intubated, bagged at 10		Temperature (T) 36.0°C
Oxygen Saturation (O_2_Sat) N/A		

**Figure:**
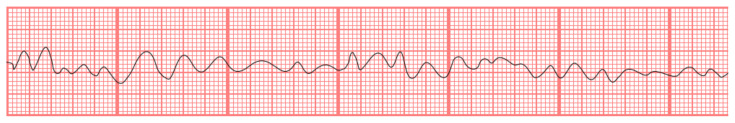


**Figure:**
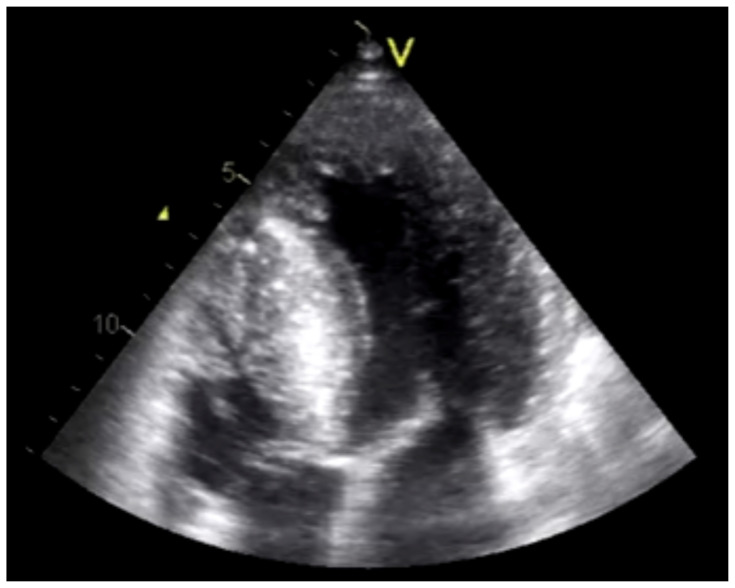


**Table:** 

*Complete blood count (CBC)*	
White blood count (WBC)	12.0 ×1000/mm^3^
Hemoglobin (Hgb)	14.0 g/dL
Platelet (Plt)	250 ×1000/mm^3^

**Table:** 

*Basic metabolic panel (BMP)*	
Sodium	140 mEq/L
Chloride	103 mEq/L
Potassium	4.5 mEq/L
Bicarbonate (HCO_3_)	20 mEq/L
Blood Urea Nitrogen (BUN)	15 mg/dL
Creatine (Cr)	1.0 mg/dL
Glucose	90 mg/dL

**Table:** 

*Extended Electrolytes*	
Calcium	9.0 mg/dL
Magnesium	2.3 mg/dL
Phosphorus	3.4 mg/dL
Albumin	4 g/dL
Thyroid Stimulating Hormone	2 milli-international units/L

**Table:** 

*Venous Blood Gas (VBG)*	
pH	7.1
PaCO2	50 mmHg
PaO2	100 mmHg
Bicarbonate (HCO_3_)	20 mEq/L

**Table:** 

*Lactic Acid*	8 mmol/L

**Table:** 

*Cardiac/Coags*	
Troponin I	0.1 ng/mL
D-dimer	< 150 ng/mL
International Normalized Ratio (INR)	1
Partial Thromboplastin Time (PTT)	30 seconds

**Table:** 

*Biliary*	
Aspartate aminotransferase (AST)	25 units/L
Alanine aminotransferase (ALT)	27 units/L
Alkaline phosphatase	75 units/L
Total bilirubin	1.0 mg/dL
Lipase	20 units/L

**Table:** 

*Toxicology*	
Ethanol	negative
Salicylates	negative
Acetaminophen	negative
Digoxin level	negative
Osmolality	285 mOsm/kg

**Table:** SIMULATION EVENTS TABLE:

Minute (state)	Participant action/trigger	Patient status (simulator response) & operator prompts	Monitor display (vital signs)
0:00 (Baseline)	Define individual roles Set up the room for a code Once roles are defined and learners are in place, then EMS arrives		T: 36 C HR: 0 BP: N/A RR: 10 bagged O2: N/A
1:00 EMS arrival	Take report from EMS Transition care/compressions	Unresponsive, cardiac arrest	T: 36 C HR: 0 BP: N/A RR: 10 bagged O2: N/A
2:00 ACLS	Verify IV access and ETT positioning Place the patient on the monitor/apply pads Continue ACLS, including chest compressions, pulse checks every 2 minutes, defibrillating every 2 minutes Administer 1mg epinephrine every 3–5 minutes Bedside ultrasound shows the heart fibrillating with hypertrophic septum Defibrillation and medications are unsuccessful in achieving ROSC	Unresponsive, cardiac arrest	T: 36 C HR: 0 BP: N/A RR: 10 bagged O2: N/A
6:00 Alternative strategies	After the 2^nd^ pulse check/defibrillation, if the learners have not attempted anything outside of the ACLS algorithm, facilitator should recommend “alternative therapies” Alternative therapies may include ECMO, double-defibrillation, stellate ganglion block, or other anti-arrhythmics such as sotalol, esmolol, lidocaine, proparacaine, etc. If the learners try any of these alternative therapies other than ECMO, the narrator should state that they have been attempted, but the patient is still in V fib.	Unresponsive, cardiac arrest	T: 36 C HR: 0 BP: N/A RR: 10 bagged O2: N/A
10:00 Recommend mechanical support	If 10 minutes go by and learners have not initiated ECMO, the facilitator should lead them in that direction without explicitly saying it. For example: “Is there any other form of mechanical cardiopulmonary support that could be considered?”	Unresponsive, cardiac arrest	T: 36 C HR: 0 BP: N/A RR: 10 bagged O2: N/A
12:00 Call consultants	If 12 minutes go by and learners have not initiated ECMO, the facilitator should recommend calling CT surgery/ICU to aid with initiating ECMO The team calls CT surgery/ICU team to present the case Consultants agree on starting ECMO and tell the ED team to start cannulating the femoral artery and femoral vein	Unresponsive, cardiac arrest	T: 36 C HR: 0 BP: N/A RR: 10 bagged O2: N/A
15:00 (Case Completion)	Case Completion/Debrief		

**Table:** 

**Critical Actions:** Team members define roles prior to EMS arrival in preparation for the code Take report from EMS and transition care to the ED team Verify IV access and ETT positioning Place the patient on the monitor Continue ACLS, including chest compressions, applying pads, defibrillating, pulse checks, and administering medications such as Epi/amio, etc, as appropriate Recognize that routine ACLS is unsuccessful in this case, and thus recommend initiating the patient on ECMO Call the appropriate consultant (cardiothoracic surgery and/or ICU) to dispo the patient, while the rest of the team starts cannulating for ECMO	0:00

**Table:** 

	Milestone	Did not achieve level 1	Level 1	Level 2	Level 3
1	**Emergency Stabilization (PC1)**	□ Did not achieve Level 1	□ Recognizes abnormal vital signs	□ Recognizes an unstable patient, requiring intervention Performs primary assessment Discerns data to formulate a diagnostic impression/plan	□ Manages and prioritizes critical actions in a critically ill patient Reassesses after implementing a stabilizing intervention
2	**Performance of focused history and physical (PC2)**	□ Did not achieve Level 1	□ Performs a reliable, comprehensive history and physical exam	□ Performs and communicates a focused history and physical exam based on chief complaint and urgent issues	□ Prioritizes essential components of history and physical exam given dynamic circumstances
3	**Diagnostic studies (PC3)**	□ Did not achieve Level 1	□ Determines the necessity of diagnostic studies	□ Orders appropriate diagnostic studies. Performs appropriate bedside diagnostic studies/procedures	□ Prioritizes essential testing Interprets results of diagnostic studies Reviews risks, benefits, contraindications, and alternatives to a diagnostic study or procedure
4	**Diagnosis (PC4)**	□ Did not achieve Level 1	□ Considers a list of potential diagnoses	□ Considers an appropriate list of potential diagnosis May or may not make correct diagnosis	□ Makes the appropriate diagnosis Considers other potential diagnoses, avoiding premature closure
5	**Pharmacotherapy (PC5)**	□ Did not achieve Level 1	□ Asks patient for drug allergies	□ Selects an medication for therapeutic intervention, consider potential adverse effects	□ Selects the most appropriate medication and understands mechanism of action, effect, and potential side effects Considers and recognizes drug-drug interactions
6	**Observation and reassessment (PC6)**	□ Did not achieve Level 1	□ Reevaluates patient at least one time during case	□ Reevaluates patient after most therapeutic interventions	□ Consistently evaluates the effectiveness of therapies at appropriate intervals
7	**Disposition (PC7)**	□ Did not achieve Level 1	□ Appropriately selects whether to admit or discharge the patient	□ Appropriately selects whether to admit or discharge Involves the expertise of some of the appropriate specialists	□ Educates the patient appropriately about their disposition Assigns patient to an appropriate level of care (ICU/Tele/Floor) Involves expertise of all appropriate specialists
9	**General Approach to Procedures (PC9)**	□ Did not achieve Level 1	□ Identifies pertinent anatomy and physiology for a procedure Uses appropriate Universal Precautions	□ Obtains informed consent Knows indications, contraindications, anatomic landmarks, equipment, anesthetic and procedural technique, and potential complications for common ED procedures	□ Determines a back-up strategy if initial attempts are unsuccessful Correctly interprets results of diagnostic procedure
20	**Professional Values (PROF1)**	□ Did not achieve Level 1	□ Demonstrates caring, honest behavior	□ Exhibits compassion, respect, sensitivity and responsiveness	□ Develops alternative care plans when patients’ personal beliefs and decisions preclude standard care
22	**Patient centered communication (ICS1)**	□ Did not achieve level 1	□ Establishes rapport and demonstrates empathy to patient (and family) Listens effectively	□ Elicits patient’s reason for seeking health care	□ Manages patient expectations in a manner that minimizes potential for stress, conflict, and misunderstanding. Effectively communicates with vulnerable populations, (at risk patients and families)
23	**Team management (ICS2)**	□ Did not achieve level 1	□ Recognizes other members of the patient care team during case (nurse, techs)	□ Communicates pertinent information to other healthcare colleagues	□ Communicates a clear, succinct, and appropriate handoff with specialists and other colleagues Communicates effectively with ancillary staff

## INSTRUCTOR MATERIALS

**Case Title:** ECMO for Refractory Cardiac Arrest

**Case Description & Diagnosis (short synopsis):** A previously healthy 22 y/o male collapses on the basketball court and is found to be in ventricular fibrillation secondary to hypertrophic obstructive cardiomyopathy (HOCM), diagnosed via ultrasound during pulse check. The standard ACLS treatments are unsuccessful in achieving ROSC. Any alternative medications are also ineffective in treating this scenario of refractory cardiac arrest. The ultimate goal for the learners and the one treatment that saves the patient is to initiate VA ECMO.

### Equipment or Props Needed

- Mannequin × 2 (one for running the code, one for cannulation)
- Endo-Circuit or lower-fidelity silicone tubing for cannulation
- ECMO cannulation kit
- Code cart including ACLS medications (epinephrine, amiodarone, procainamide, sotalol, esmolol, etc.)
- Defibrillator/pads/monitor

### Confederates needed

This simulation needs at least one confederate to act as the paramedic, who will summarize the pre-hospital events and transition care to the ED team. A narrator in the simulation control room would also be helpful. We had the learners play all other roles including team leader, airway physician, cannulating physician (all eventually get to practice this role during stage 3), nurse/techs as needed.

### Stimulus Inventory

**Background and brief information:** You are working in the ED during a busy Monday afternoon shift when you get a call from EMS stating that they are bringing in a 22-year-old male who collapsed and became pulseless while playing basketball at the gym. Compressions are in progress. ETA 5 minutes.

**Initial presentation:** EMS arrives and gives their report: “This is a 22 y/o previously healthy male who collapsed while playing basketball about 20 minutes ago. His friends checked a pulse, called 911, and immediately started chest compressions. The monitor showed V fib in the field. The patient has received 3 shocks, 2 rounds of Epi, and amiodarone is hanging. We never achieved ROSC. The monitor continues to show V fib. We successfully intubated the patient in the field using a 7.5 ETT, currently 22 cm at the lip. Initial blood glucose was 90. What else would you like to know?”

### How the scene unfolds

- The goal is to push the learners towards initiating ECMO. Normal ACLS algorithm is unsuccessful.
- If learners continue ACLS algorithm for more than 5 minutes, the narrator in the control room should prompt them to try alternative ideas.
- Double defibrillation, stellate ganglion block, and other anti-arrhythmics such as sotalol/lidocaine/esmolol are all unsuccessful. If the learners mention these, the narrator should state that they have been attempted and are unsuccessful.
- If the learners have not started ECMO at the 10-minute mark, the narrator should push them toward ECMO without overtly saying it, such as, “Is there any other form of mechanical cardiopulmonary support that could be considered?”
- If the learners have still not started initiating ECMO at the 12-minute mark, the narrator should give them the direct recommendation.
- If at any point the learners mention ending the resuscitation, the narrator should interject: “But doctors, the patient is so young and otherwise healthy; there has to be another life-supporting strategy we can try.”
- Once the learners suggest ECMO, they should call either the cardiothoracic surgery team and/or the ICU team. The learners should give a quick presentation over the phone of the current clinical situation and treatments that have already been attempted. The learner should recommend ECMO to the consultant. The consultant agrees and asks the ED team to start cannulating the patient for VA ECMO. This ends stage 1.
- Stage 2 involves debriefing the simulation of the refractory cardiac arrest. Have the learners reflect about how the code was run. Were roles assigned appropriately? How was team communication? How was the medical management of the code? What did they do well? What could be improved upon?
- Stage 3 involves a brief overview of ECMO, including the basic concept of how ECMO works physiologically, highlighting the differences between VV and VA ECMO, and the indications/contraindications to starting ECMO. The printed handout may be provided to the learners at this time. This stage also involves a step-by-step demonstration of the cannulation process to the learners.
- Stage 4 involves hands-on learning, having the trainees practice the cannulation procedure themselves.

### Critical actions

1. Team members define roles prior to EMS arrival, in preparation for the code
2. Take report from EMS and transition care to the ED team
3. Verify IV access and ETT (endotracheal tube) positioning
4. Place the patient on the monitor
5. Continue ACLS, including chest compressions, applying pads, defibrillating, pulse checks, and administering medications such as Epi/amio, etc, as appropriate
6. Recognize that routine ACLS is unsuccessful in this case and thus recommend initiating the patient on ECMO
7. Call the appropriate consultant (cardiothoracic surgery and/or ICU) to disposition the patient, while the rest of the team starts cannulating for ECMO

**Case Title:** ECMO for Refractory Cardiac Arrest

**Chief Complaint:** Cardiac Arrest

**General Appearance:** comatose, pulseless with active compressions, intubated, being bagged

### Primary Survey

- **Airway:** intubated with 7.5 ETT, measuring 22cm at the lip; tube condensates with each breath
- **Breathing:** clear breath sounds bilaterally when bagged without wheezes/rales
- **Circulation:** pulseless, but 2+ femoral pulse can be palpated bilaterally during compressions

### History

- **History of present illness:** 22 y/o previously healthy male, collapsed while playing basketball. His friends checked for a pulse, which was absent. They immediately started chest compressions and called 911. Monitor showed V fib in the field, and EMS successfully intubated the patient. Glucose in the field was 90. Patient has received a total of 3 shocks, 2 rounds of Epi, and amiodarone is hanging prior to arrival to the ED. EMS was unable to obtain ROSC.
- **Past medical history:** none
- **Past surgical history:** none
- **Patients medications:** none
- **Allergies:** none
- **Social history:** the patient attends college. He drinks socially on the weekends with occasional marijuana use. No tobacco use. No other illicit drug use.
- **Family history:** the patient’s uncle died at a young age due to unknown reasons

### Secondary Survey/Physical Examination

- **General appearance:** comatose, pulseless with active compressions, intubated, being bagged
- **HEENT:** intubated
  ○ **Head:** within normal limits
  ○ **Eyes:** within normal limits
  ○ **Ears:** within normal limits
  ○ **Throat:** within normal limits
- **Neck:** within normal limits
- **Heart:** absent heart sounds
- **Lungs:** clear breath sounds bilaterally when bagged without wheezes/rales
- **Abdominal/GI:** within normal limits
- **Genitourinary:** within normal limits
- **Rectal:** within normal limits
- **Extremities:** within normal limits
- **Back:** within normal limits
- **Neuro:** comatose, Glasgow Coma Scale 3T
- **Skin:** within normal limits
- **Lymph:** within normal limits
- **Psych:** N/A

### Results

*Cardiac monitor* *11*

Chikumaya, A drawn image of ventricular fibrillation on II-lead EKG. In: *Wikimedia Commons*. https://commons.wikimedia.org/wiki/File:V_f.png. Public domain.

*Echo: heart is fibrillating* *12*

CardioNetworks. HOCM with SAM. In: *Wikimedia Commons*.

https://commons.wikimedia.org/wiki/File:HOCM_with_SAM_E00515_(CardioNetworks_ECHOpedia).webm. CC BY-SA 3.0

*Lab Work:*

## OPERATOR MATERIALS

### Diagnosis

Refractory ventricular fibrillation secondary to previously undiagnosed hypertrophic obstructive cardiomyopathy (HOCM).

### Disposition

Disposition to the ICU after ECMO cannulation.

## DEBRIEFING AND EVALUATION PEARLS

### ECMO for Refractor Cardiac Arrest

#### Teaching Pearls

- Refractory cardiac arrest is defined as the lack of ROSC after 10–30 minutes of appropriate CPR.^1^,^2^ Refractory ventricular fibrillation has slightly differing definitions, including VF persisting despite 3 shocks, or the combination of 3 unsuccessful shocks plus amiodarone.^1^,^3^
- Remember the “H’s and T’s” as causes of cardiac arrest. These are especially important to consider when having difficulty achieving ROSC.^4^
  - Hypoxia, Hypovolemia, Hypothermia, Hyper/Hypokalemia, Hydrogen Ions (Acidosis)
  - Tension pneumothorax, Tamponade, Toxins, Thrombosis (coronary and pulmonary)
- ECPR is the utilization of VA ECMO for the patient in cardiac arrest

##### Indications for ECMO

- Acute, severe respiratory or cardiac failure
- REVERSIBLE cause
- Refractory to usual treatment
- High risk of death
- May act as a bridge for definitive therapy, such as VAD (ventricular assist device) or transplant

##### Contraindications

- DNR/DNI (do not resuscitate/do not intubate)
- Irreversible process
- Unwitnessed cardiac arrest
- Advanced malignancy

##### VV ECMO is used for acute respiratory failure. Specific indications include

- ARDS (acute respiratory distress syndrome)
- Smoke inhalation/airway obstruction
- Status asthmaticus
- Pulmonary contusion
- Pulmonary hemorrhage/massive hemoptysis
- Meconium aspiration
- Lung transplant failure
- Bridge to lung transplant

##### VA ECMO is primarily used for acute cardiac failure, but also supports acute respiratory failure. Specific indications include

- Cardiac arrest
- Cardiogenic shock
- ACS (acute coronary syndrome)
- Arrhythmia
- PE (pulmonary embolism)
- Heart transplant failure
- Bridge to LVAD (left ventricular assist device)

##### Choosing the appropriate site to cannulate

- VV: Cannulate the internal jugular (IJ) and femoral vein, or can do one double-lumen catheter in the right IJ, but need transesophageal echocardiogram (TEE) or fluoroscopy.
- VA: Cannulate the venous system at the internal jugular or femoral vein. The arterial system is most commonly cannulated via the femoral artery, less commonly carotid or direct surgical cutdown.

##### The cannulation process

- The cannulation procedure is similar to placing a central line or arterial line, utilizing Seldinger technique. The main difference includes the additional serial dilations to accommodate the large ECMO catheters.
- Pay attention to the size of your ECMO catheters, particularly in regard to dilation. Tubing size will vary from institution to institution, but in general the venous catheter will be larger than the arterial catheter. The last dilator you use prior to inserting the ECMO catheter should be 1 size SMALLER than the catheter size.
- The tip of the venous catheter should approximate the opening of the right atrium. This can be verified utilizing bedside ultrasound. The arterial catheter is inserted until the metal ring near the distal end of the catheter approximates the skin.
- Once the ECMO catheters are in place, clamp the distal ends utilizing Kelly forceps, pour sterile saline into the distal ends of the catheters to displace any air in the tubing. Then carefully connect the ECMO catheters to the primed circuit tubing, making sure there are no air bubbles in the system. You are now ready to start the ECMO circuit.

##### Complications

- Bleeding: 30–50% of patients^5^,^6^
- Thromboembolism: up to 16% of pts^5^
- Pulmonary edema leading to hemorrhage (VA)
- Cardiac Thrombosis (VA)
- Coronary and/or Cerebral ischemia (VA)

##### Outcomes

- Average survival to hospital discharge for out of hospital cardiac arrest without using ECMO is only about 10%^7^
- Data varies, but neurological-intact survival utilizing ECPR ranges from 12% all the way up to 54%^3^,^8^–^11^

*These above teaching pearls may be printed as a hand-out for the end of the simulation*.

## SIMULATION ASSESSMENT

*ECMO for Refractory Cardiac Arrest*

Learner: _________________________________________

***Assessment Timeline***

This timeline is to help observers assess their learners. It allows observer to make notes on when learners performed various tasks, which can help guide debriefing discussion.

**Critical Actions:**

□ Team members define roles prior to EMS arrival in preparation for the code
□ Take report from EMS and transition care to the ED team
□ Verify IV access and ETT positioning
□ Place the patient on the monitor
□ Continue ACLS, including chest compressions, applying pads, defibrillating, pulse checks, and administering medications such as Epi/amio, etc, as appropriate
□ Recognize that routine ACLS is unsuccessful in this case, and thus recommend initiating the patient on ECMO
□ Call the appropriate consultant (cardiothoracic surgery and/or ICU) to dispo the patient, while the rest of the team starts cannulating for ECMO

**Summative and formative comments:**

**Milestones assessment:**
