# Supplementary material for: Extracorporeal Membrane Oxygenation (ECMO) for Refractory Cardiac Arrest
Source: J Educ Teach Emerg Med. 2020 Oct 15;5(4):S28–58. doi: 10.21980/J88W69 (PMC10332526; doi:10.21980/J88W69)
Supplement: Supplementary file 1 [file jetem-5-2-s28-supp1.pptx]

## Slide 1
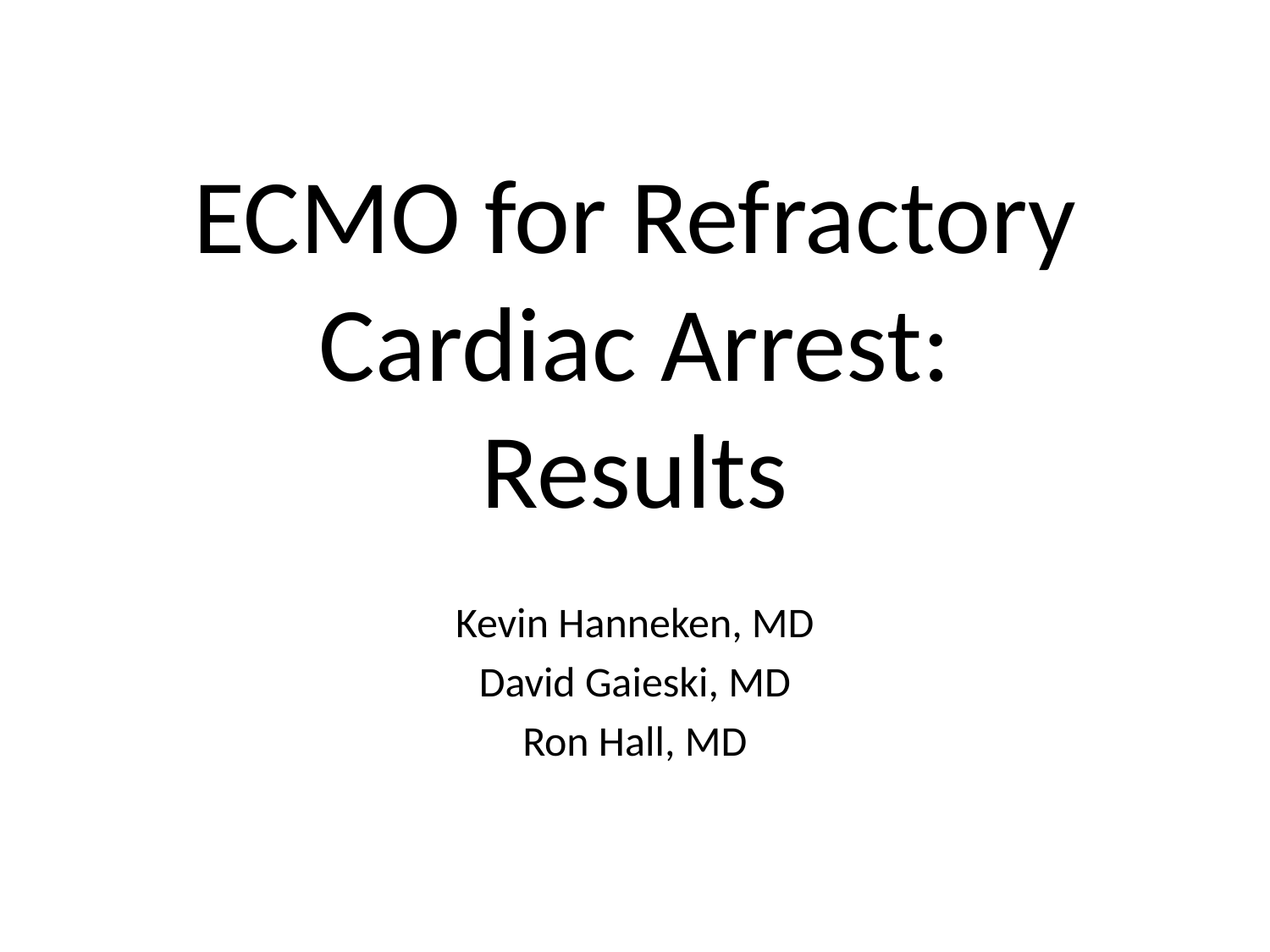

# ECMO for Refractory Cardiac Arrest: Results
Kevin Hanneken, MD
David Gaieski, MD
Ron Hall, MD

## Slide 2
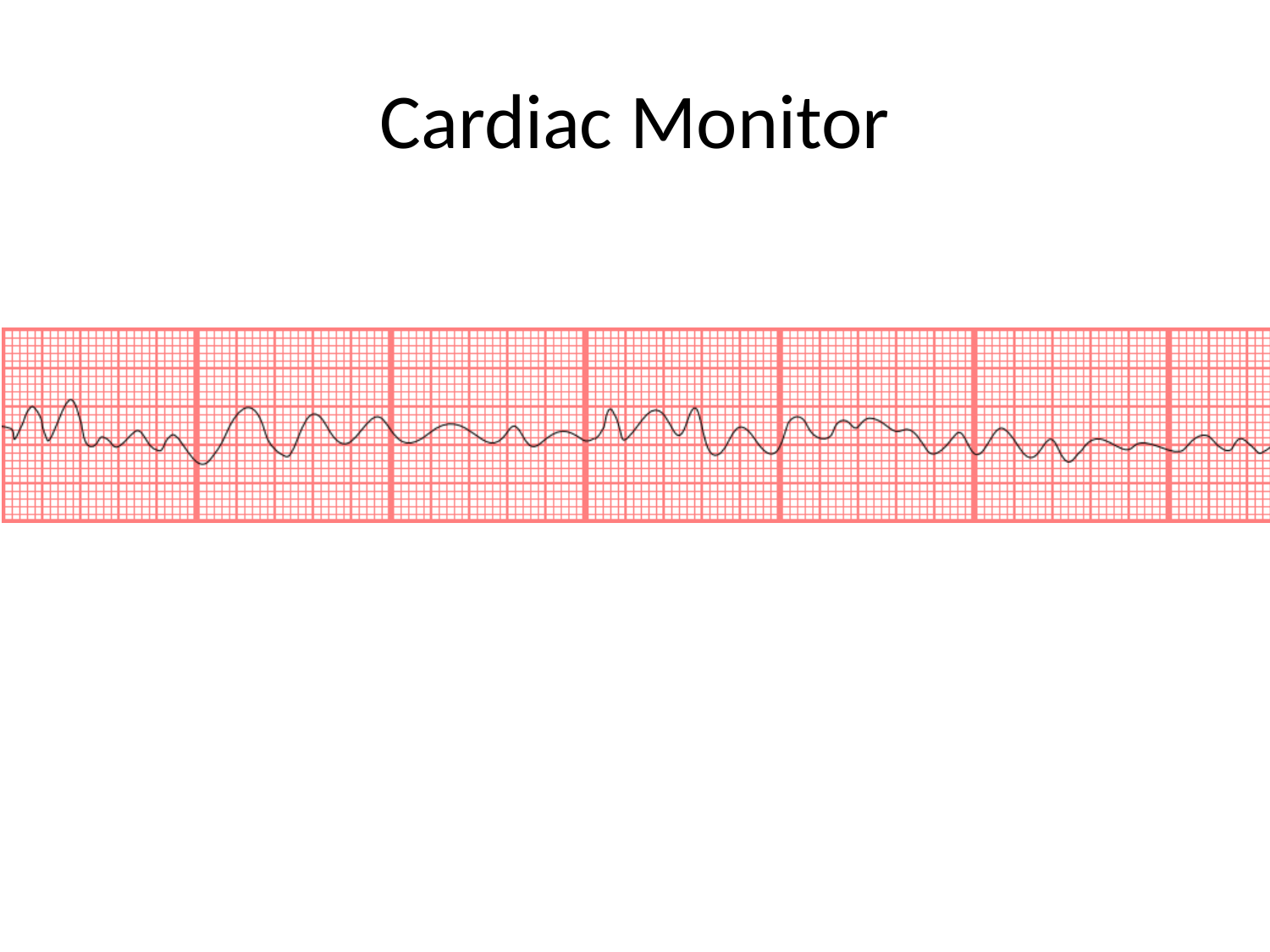

# Cardiac Monitor

## Slide 3
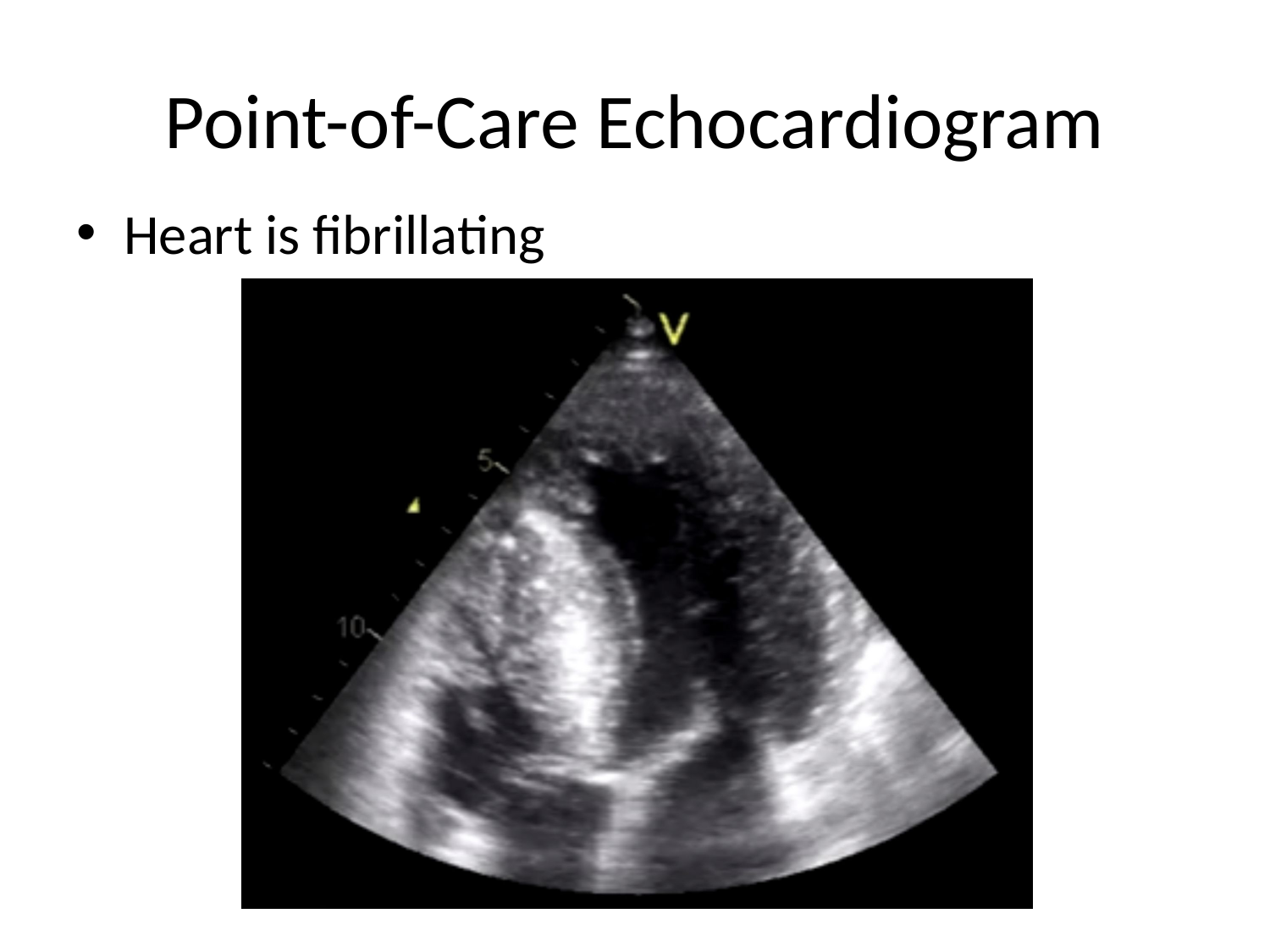

# Point-of-Care Echocardiogram
Heart is fibrillating

## Slide 4
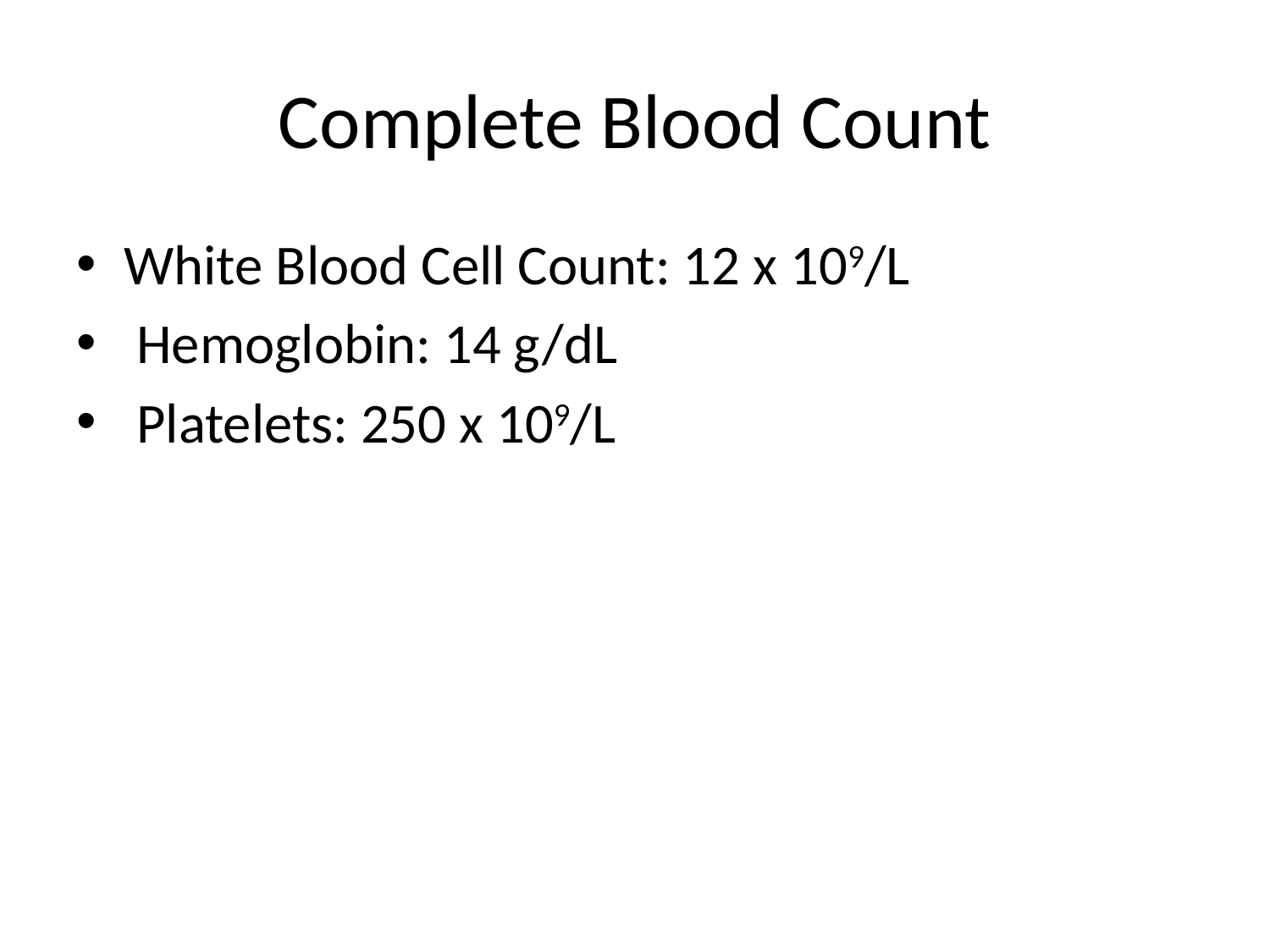

# Complete Blood Count
White Blood Cell Count: 12 x 109/L
 Hemoglobin: 14 g/dL
 Platelets: 250 x 109/L

## Slide 5
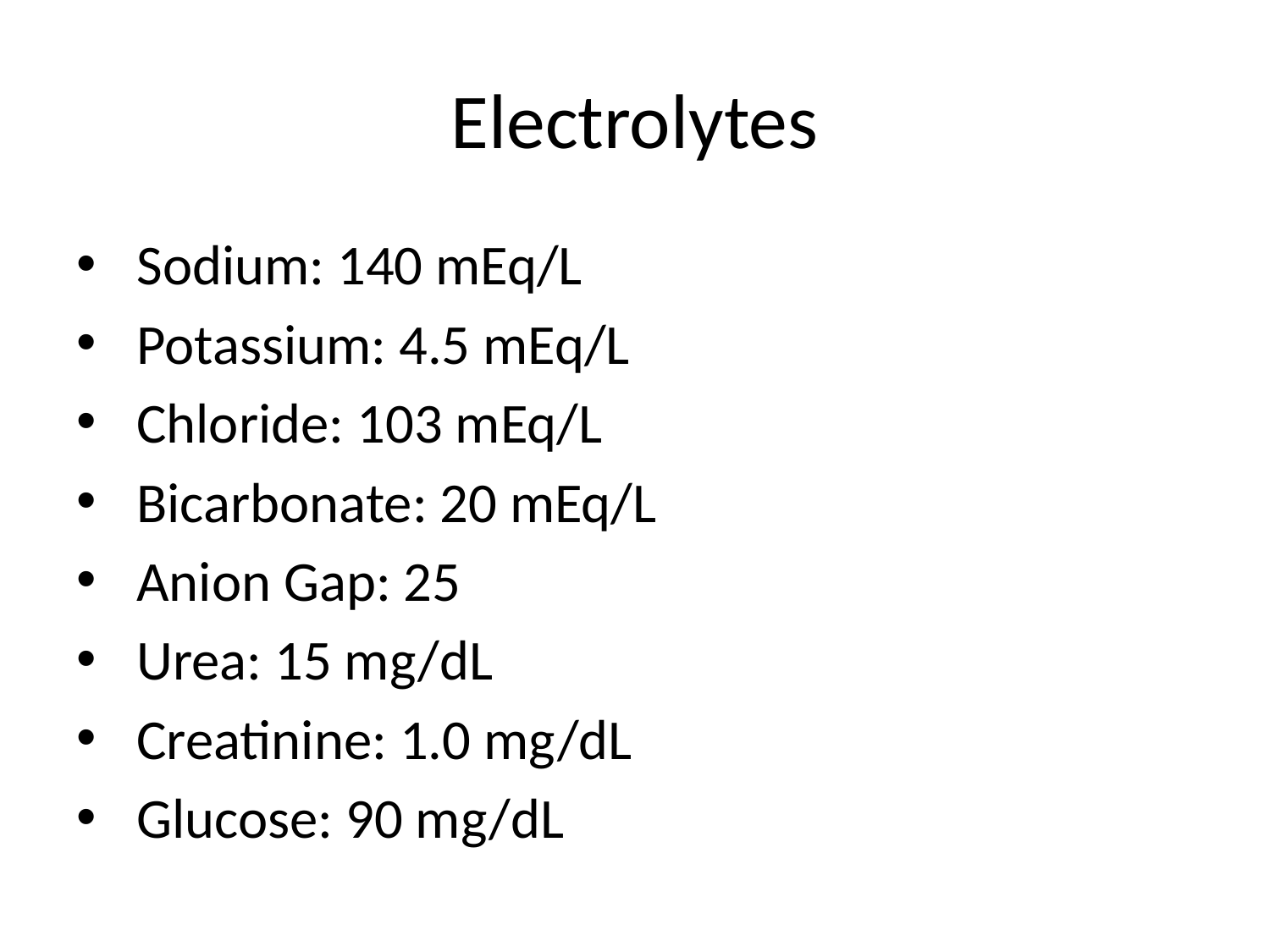

# Electrolytes
 Sodium: 140 mEq/L
 Potassium: 4.5 mEq/L
 Chloride: 103 mEq/L
 Bicarbonate: 20 mEq/L
 Anion Gap: 25
 Urea: 15 mg/dL
 Creatinine: 1.0 mg/dL
 Glucose: 90 mg/dL

## Slide 6
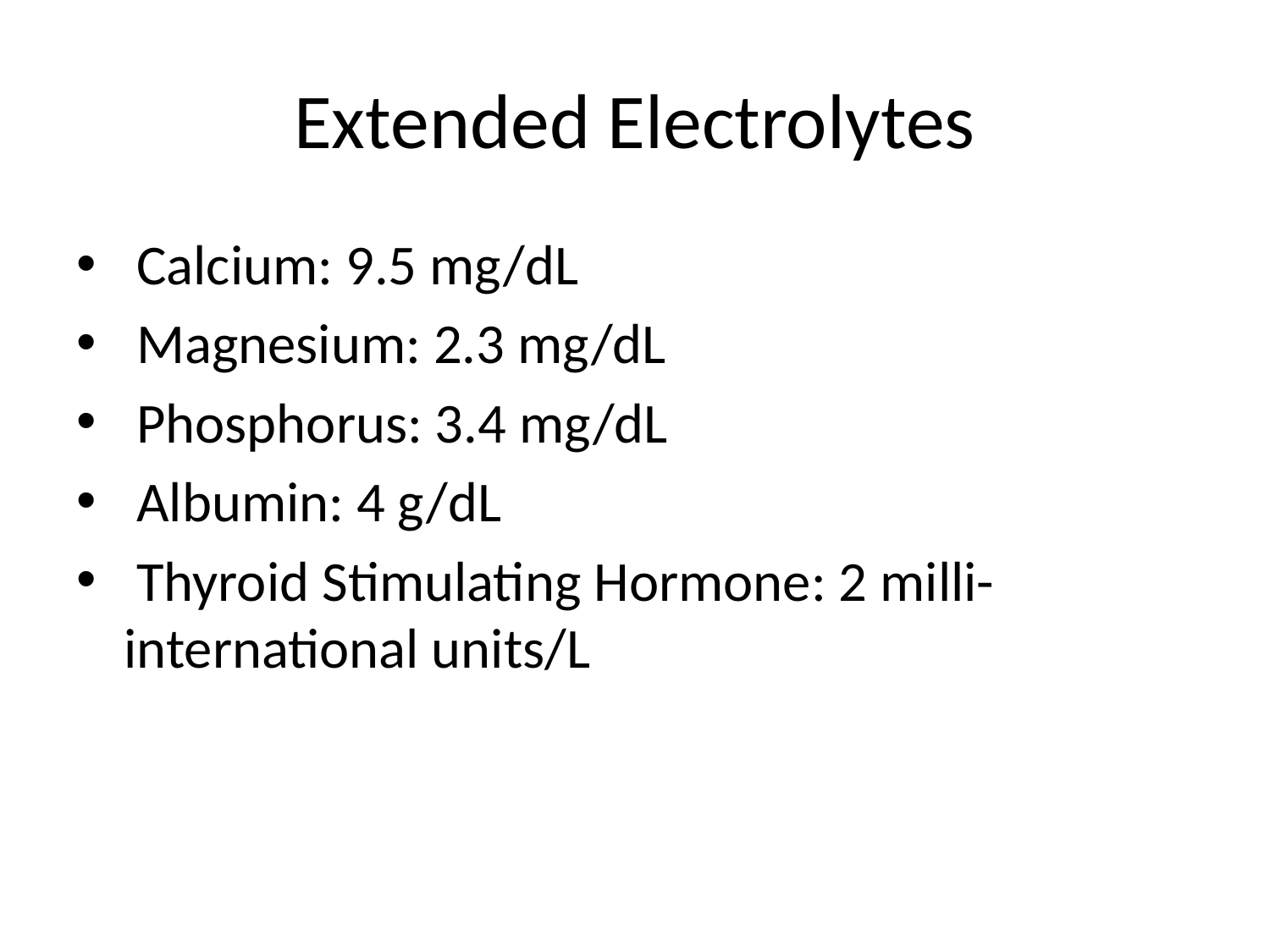

# Extended Electrolytes
 Calcium: 9.5 mg/dL
 Magnesium: 2.3 mg/dL
 Phosphorus: 3.4 mg/dL
 Albumin: 4 g/dL
 Thyroid Stimulating Hormone: 2 milli-international units/L

## Slide 7
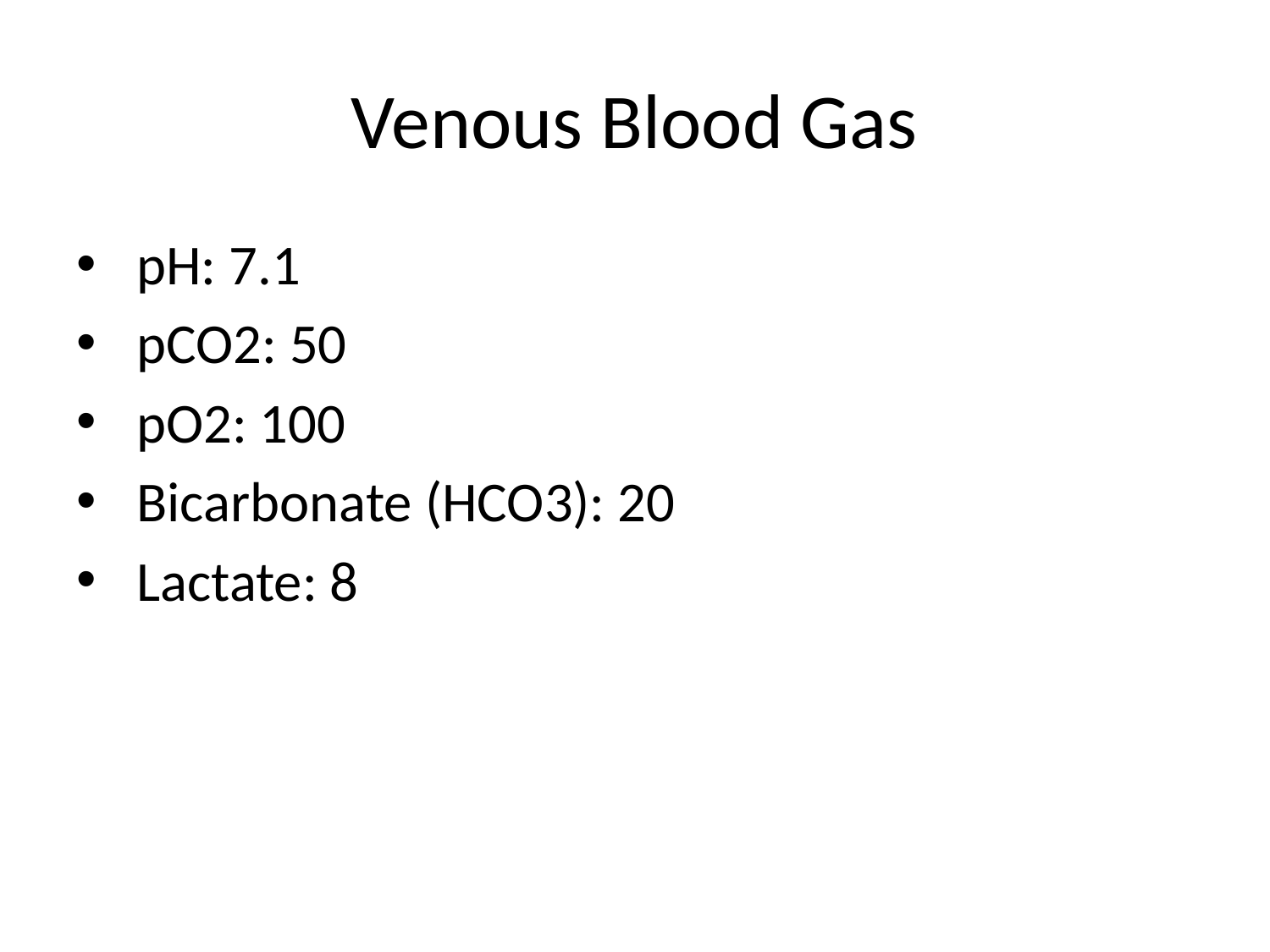

# Venous Blood Gas
 pH: 7.1
 pCO2: 50
 pO2: 100
 Bicarbonate (HCO3): 20
 Lactate: 8

## Slide 8
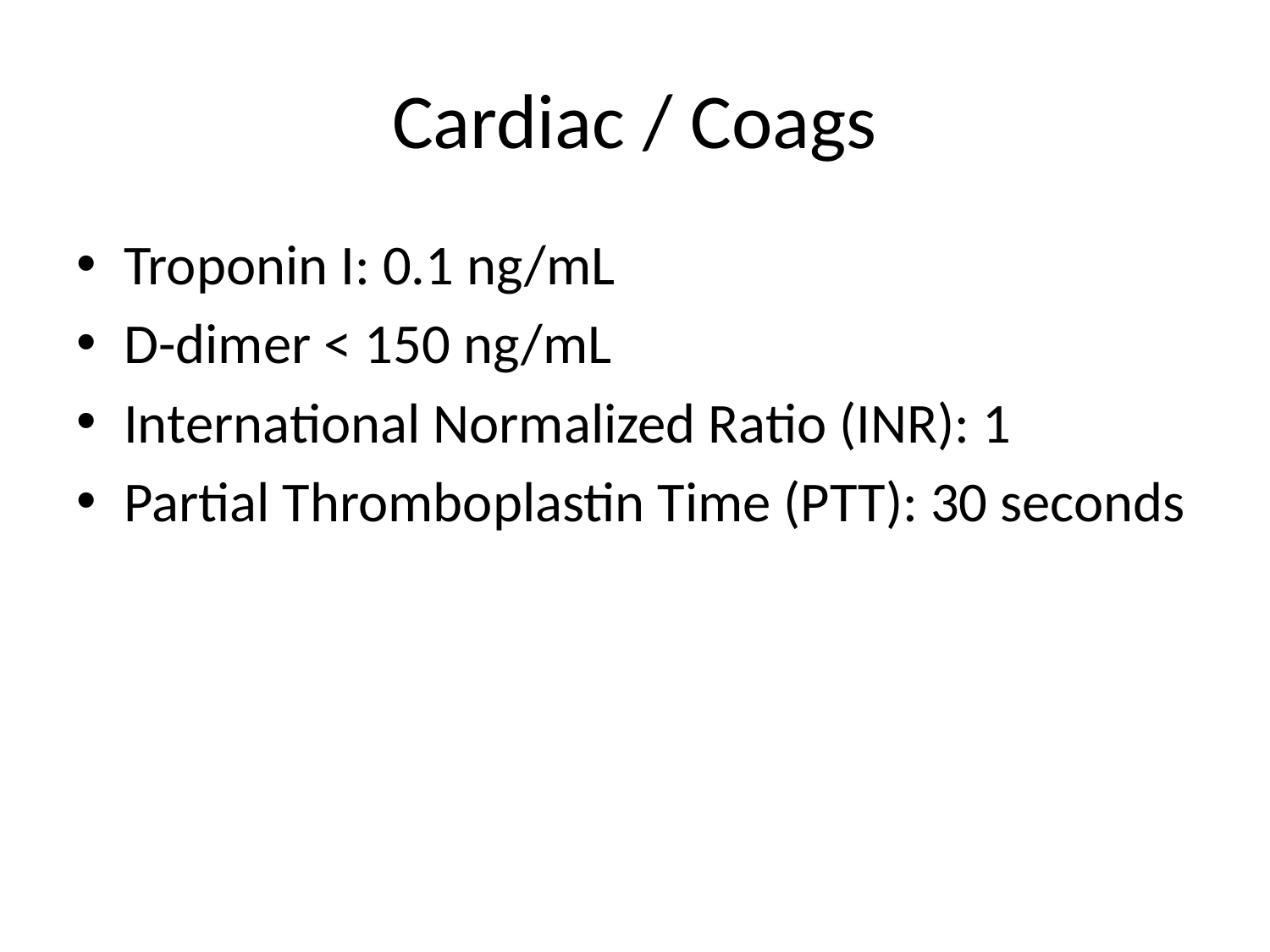

# Cardiac / Coags
Troponin I: 0.1 ng/mL
D-dimer < 150 ng/mL
International Normalized Ratio (INR): 1
Partial Thromboplastin Time (PTT): 30 seconds

## Slide 9
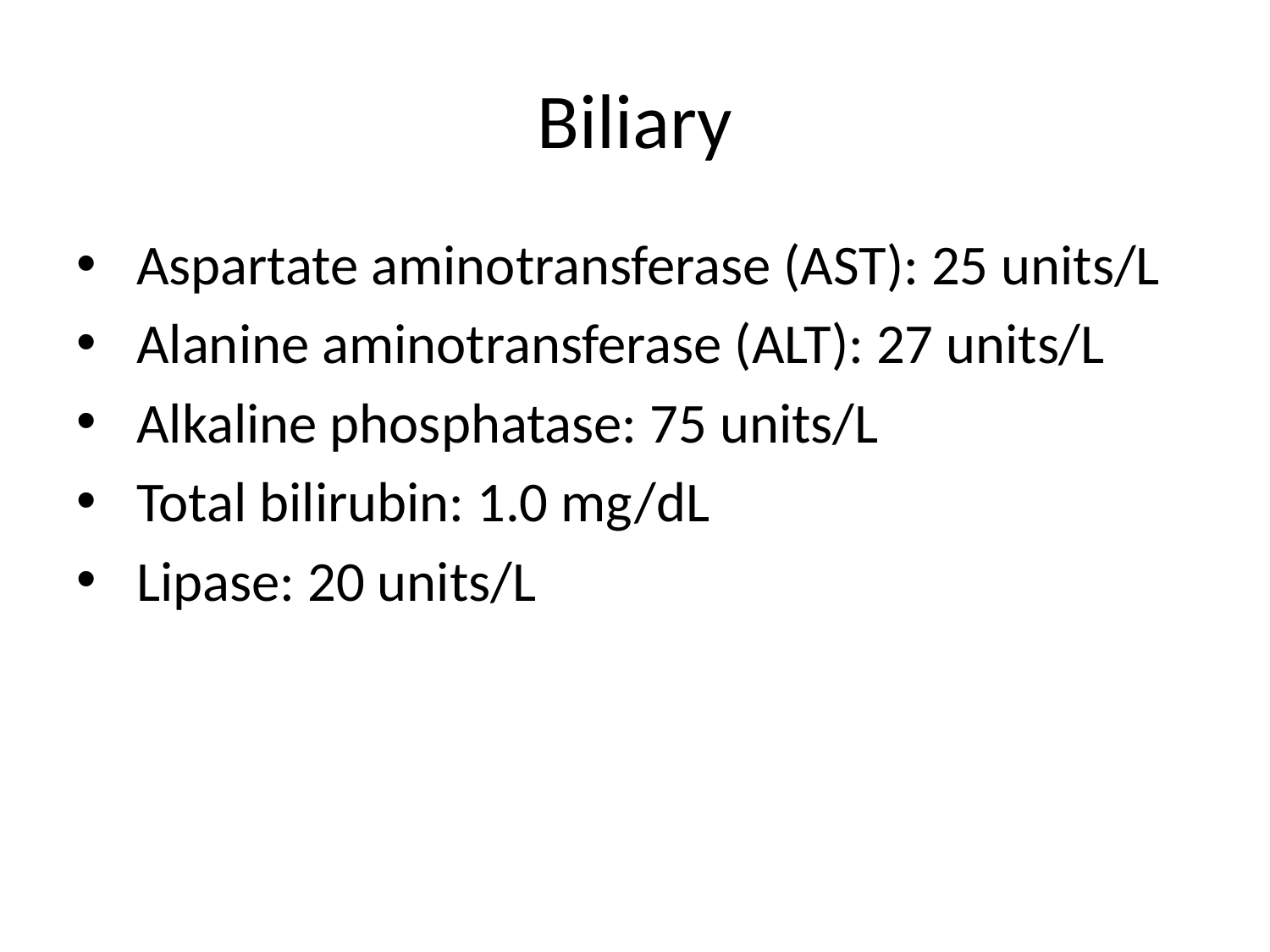

# Biliary
 Aspartate aminotransferase (AST): 25 units/L
 Alanine aminotransferase (ALT): 27 units/L
 Alkaline phosphatase: 75 units/L
 Total bilirubin: 1.0 mg/dL
 Lipase: 20 units/L

## Slide 10
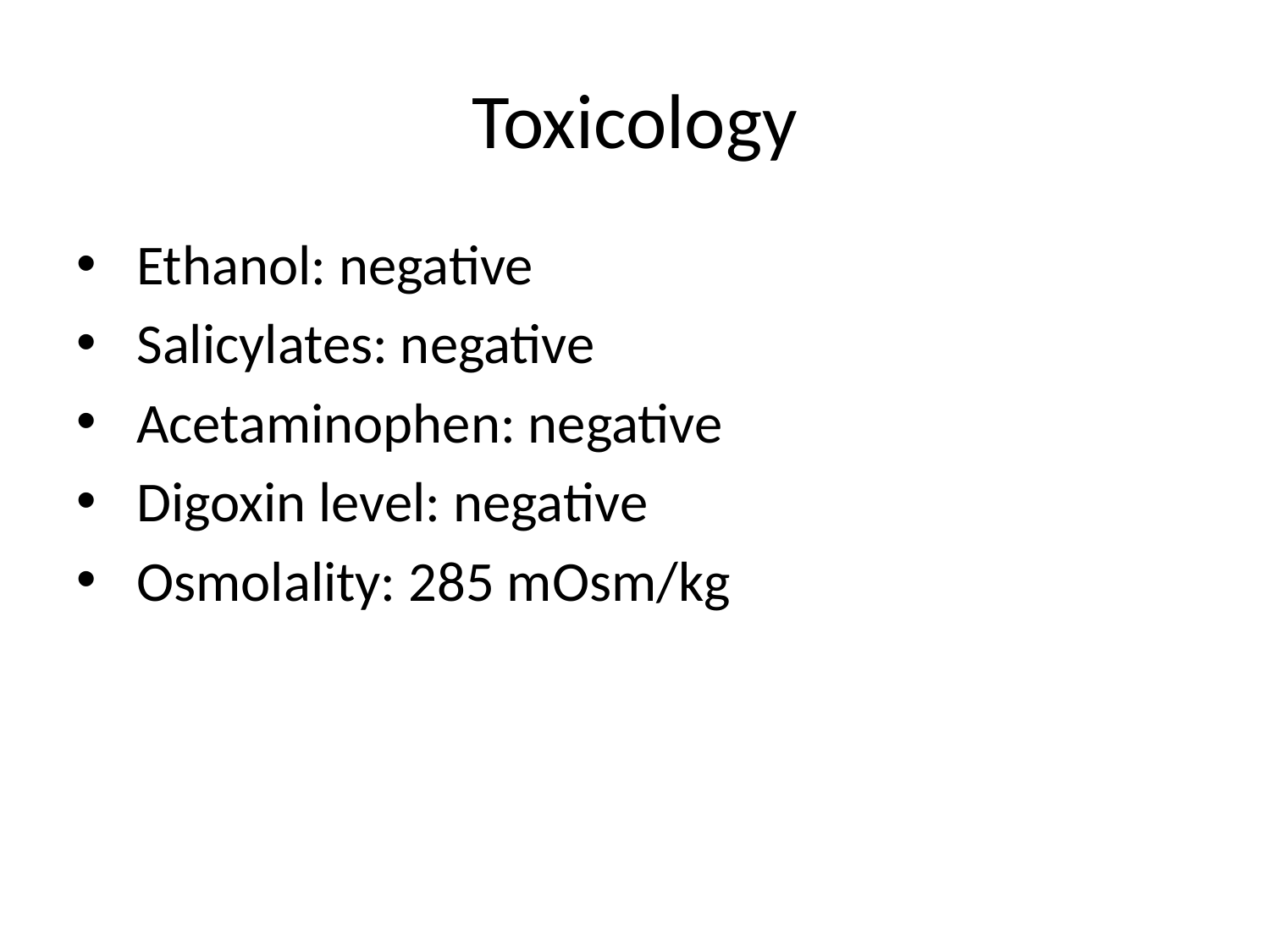

# Toxicology
 Ethanol: negative
 Salicylates: negative
 Acetaminophen: negative
 Digoxin level: negative
 Osmolality: 285 mOsm/kg
